# Plant Microbiome and Its Link to Plant Health: Host Species, Organs and *Pseudomonas syringae* pv. *actinidiae* Infection Shaping Bacterial Phyllosphere Communities of Kiwifruit Plants

**DOI:** 10.3389/fpls.2018.01563

**Published:** 2018-11-07

**Authors:** Witoon Purahong, Luigi Orrù, Irene Donati, Giorgia Perpetuini, Antonio Cellini, Antonella Lamontanara, Vania Michelotti, Gianni Tacconi, Francesco Spinelli

**Affiliations:** ^1^Department of Soil Ecology, Helmholtz Center for Environmental Research - UFZ, Halle, Germany; ^2^CREA Research Centre for Genomics and Bioinformatics – Fiorenzuola d’Arda, Italy; ^3^Department of Agricultural and Food Sciences, Alma Mater Studiorum – Università di Bologna, Bologna, Italy

**Keywords:** *Actinidia chinensis*, *Actinidia deliciosa*, epiphytic community, metagenome, bacterial biocoenosis, biocontrol, bacterial canker

## Abstract

*Pseudomonas syringae* pv. *actinidiae* (Psa) is the causal agent of the bacterial canker, the most devastating disease of kiwifruit vines. Before entering the host tissues, this pathogen has an epiphytic growth phase on kiwifruit flowers and leaves, thus the ecological interactions within epiphytic bacterial community may greatly influence the onset of the infection process. The bacterial community associated to the two most important cultivated kiwifruit species, *Actinidia chinensis* and *Actinidia deliciosa*, was described both on flowers and leaves using Illumina massive parallel sequencing of the V3 and V4 variable regions of the 16S rRNA gene. In addition, the effect of plant infection by Psa on the epiphytic bacterial community structure and biodiversity was investigated. Psa infection affected the phyllosphere microbiome structures in both species, however, its impact was more pronounced on *A. deliciosa* leaves, where a drastic drop in microbial biodiversity was observed. Furthermore, we also showed that Psa was always present in syndemic association with *Pseudomonas syringae* pv. *syringae* and *Pseudomonas viridiflava*, two other kiwifruit pathogens, suggesting the establishment of a pathogenic consortium leading to a higher pathogenesis capacity. Finally, the analyses of the dynamics of bacterial populations provided useful information for the screening and selection of potential biocontrol agents against Psa.

## Introduction

The plant microbiome plays a crucial role in plant health and productivity and, thus, has received significant attention in recent years (Turner et al., 2013). The main focuses of the plant microbiome studies are devoted to model plants, such as *Arabidopsis thaliana*, as well as important economic crop species including barley (*Hordeum vulgare*), corn (*Zea mays*), rice (*Oryza sativa*), soybean (*Glycine max*), wheat (*Triticum aestivum*), whereas less attention is given to fruit crops and tree species (Busby et al., 2017). Plant microbiomes are shaped by both plant-related (i.e., genotype, organ, species, health status etc.) and environmental factors (i.e., management, land use and climate) (Bringel and Couée, 2015). Although plant health status is reported in some studies to be reflected or linked to its microbiome (Berendsen et al., 2012; Turner et al., 2013; Berg et al., 2014), this aspect is actually still unclear and requires further empirical evidence. Thus, in fruit crop species, it is still uncertain how infectious diseases alter the microbiome of the infected organs.

*Pseudomonas syringae* pv. *actinidiae* (Psa) is the causal agent of the bacterial canker of kiwifruit, which is the major threat to kiwifruit production worldwide (Scortichini et al., 2012; Vanneste, 2012). The pathogen can infect both *Actinidia chinensis* and *A. deliciosa* plants, the two most important commercial species (Donati et al., 2014). So far, no resistant genotype has been found, but, generally, *A. deliciosa* varieties are considered less susceptible than the ones belonging to *A. chinensis* (Spinelli et al., 2011). Before infecting the plant, the pathogen grows on the epiphytic surfaces of *Actinidia* flowers and leaves. After this epiphytic phase, infection occurs via natural opening such stomata on leaves or stylar tissues on flowers (Donati et al., 2018), or via natural wounds, such as broken trichomes (Spinelli et al., 2011). Once Psa enters the host tissues, the infection rapidly becomes systemic, leading to the death of the host plant (Renzi et al., 2012; Scortichini et al., 2012; Donati et al., 2014). Therefore, the understanding of Psa interactions with the phyllosphere microbial community could provide essential information for developing innovative, effective and long-lasting control strategies. To date, no sustainable and completely effective control methods have been developed for this disease, and control mainly relies on the use of copper formulates (Collina et al., 2016; Scortichini, 2016). However, the increasing concerns about the environmental risks caused by the widespread use of xenobiotic pesticides led institutions, such as the European Commission, to develop regulations to restrict their use (Commission Regulation [EC], 2008. 396/2005/EC, 149/2008/EC). A sustainable and environment-friendly alternative to chemical pesticides for controlling disease in the phyllosphere is the use of biological control agents (BCAs) (Wicaksono et al., 2018). Indeed, the phyllosphere represents an ecological niche with pivotal agricultural and biological significance (Whipps et al., 2008; Vorholt, 2012), and the bacterial epiphytic community can positively impact plant health, physiology and environmental fitness (Kim et al., 2011; Vorholt, 2012; Dees et al., 2015). Several epiphytic bacterial species isolated from the phyllosphere have been reported to be strong competitors against plant pathogens, thus acting as BCAs (Volksch and May, 2001). In addition, to the direct competition for limited space and nutrients, some BCAs can also inhibit pathogen growth by secreting antimicrobial compounds (e.g., *Pantoea agglomerans, Lactobacillus plantarum*), or interfering with the pathogen signalling system (Volksch and May, 2001). Finally, other epiphytic bacteria are known to exert a plant growth-promoting activity and induce natural plant resistance against pathogens (Ryu et al., 2003; Ottesen et al., 2013; Rastogi et al., 2013). For the control of Psa, strains of *Pseudomonas fluorescens, Bacillus subtilis, Bacillus amyloliquefaciens* and, more recently, *Lactobacillus plantarum* have been tested as possible BCAs (Gould et al., 2015; Collina et al., 2016; Yakhin et al., 2017).

Screening and selection of new BCAs has been mainly focused on the identification of single bacterial species effective in contrasting a specific pathogen. However, under natural conditions, bacteria live in communities regulated by interspecies signalling (Ryan and Dow, 2008) and, thus, the modern approach to enhance plant growth and health is to elucidate the effect of small microbial consortia against pathogens or on plant host resistance induction (Sarma et al., 2015). Several studies highlighted that, in comparison to the use of single beneficial species, the application of microbial consortia may improve efficacy, reliability and consistency of the growth and health promotion under a wider range of environmental conditions (Stockwell et al., 2011). In this view, the beneficial effect on plant health is the result of the combined and synergic interaction of multiple bacterial species each with specific positive effect (Kim et al., 2011; Sarma et al., 2015).

Understanding the dynamics and evolution of the bacterial community on the phyllosphere may also provide crucial information on other factors influencing Psa infection process. In fact, symbiotic interactions among different microbial species, leading to a pathogenic consortium, may increase disease incidence and development (Lamichhane and Venturi, 2015). Growing evidence highlighted that pathogens do not operate independently, but their virulence is mediated by their interaction with other pathogens (Singer, 2010; Lamichhane and Venturi, 2015). This phenomenon has led researchers to develop the idea of pathobiome, i.e., a community in which pathogens participate in complex interactions with their biotic environment (Vayssier-Taussat et al., 2014). The importance of the interactions among pathogens is well recognised in human health (Singer, 2010). For example, in the medical field, the term syndemic indicates the synergistic interactions among diseases (Singer and Clair, 2003). Even though some cases of bacterial pathogens co-occurrence has been described in plants, the impact of pathogens interactions on plants diseases has received far less attention (Kùdela et al., 2010; Lamichhane and Venturi, 2015). In kiwifruit, *Pseudomonas* pathogens, such as *P. syringae* pv. *syringae* and *P. viridiflava*, often occur together but their interaction is still unclear (Balestra et al., 2008; Petriccione et al., 2017).

The main aims of this study were (i) to investigate the bacterial phyllosphere communities on leaves and flowers of two species of kiwifruit species (*A. deliciosa* cv. Hayward and *A. chinensis* cv. Hort16A) using Illumina sequencing of the V3 and V4 variable regions of the 16S ribosomal gene; (ii) to verify the relative importance of plant species, organ and Psa infection in shaping bacterial phyllosphere communities; (iii) to quantify (by quantitative real-time polymerase chain reaction, qPCR) the abundance in different plant organs of *Pseudomonas* pathogens (i.e., Psa, *P. syringae* pv. *syringae* and *P. viridiflava*) and BCAs (i.e., *Lactobacillus plantarum, Pantoea agglomerans, Bacillus subtilis, B. amyloliquefaciens*, and *P. fluorescens*) in relation to plant health status. The experimental approach allowed us to highlight the possible contribute of the species-specific microbiome on the different susceptibility of *A. deliciosa* cv. Hayward and *A. chinensis* cv. Hort16A to Psa.

## Materials and Methods

### Sample Collection

Leaves and flowers were collected from *A. deliciosa* cv. Hayward and *A. chinensis* cv. Hort16A plants grown in commercial orchards located in Faenza region (Emilia Romagna, Italy). In those orchards, the average disease incidence in the previous season were 8 and 21% in Hayward and Hort16A, respectively. At shoot emergence and beginning of blooming, an extensive screening was performed to discriminate uninfected from infected plants. For this purpose, 10 flowers and 10 leaves per each plant were sampled and Psa contamination was assessed according to Gallelli et al. (2014). The study of the microbial community in the phyllosphere was performed separately on uninfected from infected plants. For this purpose, sampling was performed at full blooming for both kiwifruit species. Standard orchard pruning, fertilisation and irrigation were applied, and no chemical or biological pesticides were applied in the 6 months preceding sampling. Moreover, the orchards were naturally pollinated, with no assisted pollen application. Leaves were sampled in groups of 10 per plant, randomly chosen either in uninfected or infected vines. To minimise the effect of leaf position and age, only the third fully expanded leaf from each shoot of the same age was collected. Flowers were sampled at anther dehiscence in groups of 5 per plant, randomly selected either in uninfected or infected vines. Each leaf or flower sample was washed with 10 or 15 ml of sterile MgSO_4_ 10 mM solution, respectively. To concentrate the bacterial load, the same washing solution was used for all samples inside a specific group. To extract all bacteria associated with phyllosphere, washing was carried out for 15 min under gentle agitation (100 rpm) at 4°C temperature to avoid mechanical tissue damage and bacterial multiplication. To verify the efficacy of the washing process, each washed leaf or flower was transferred in a new sterile solution of MgSO_4_ and processed again as previously described. This washing solution was successively plated on LB-agar medium. Finally, the grouping of the different samples according to the presence of absence of Psa was confirmed by homogenising each group of leaves or flowers in a batch and processing according to Gallelli et al. (2014).

### DNA Extraction and Sequencing

Libraries were prepared using the Illumina (San Diego, CA, United States) 16S metagenomic sequencing library preparation protocol, which allows the sequencing of the variable V3 and V4 regions of the 16S rRNA gene. Briefly, washing solutions were pelleted by centrifugation at 20,000 × *g* for 20 min at 4°C, then the supernatant was discarded. Pellets were joined, immediately frozen in liquid nitrogen and stored at -80°C. From frozen pellets, genomic DNA was extracted and purified using NucleoSpin^®^ soil kit (Macherey-Nagel GmbH & Co. KG, Düren, Germany) following manufacturer instruction. After determining its concentration and purity by spectrophotometer, the extracted genomic DNA was used as template for V3–V4 regions amplification with 16S Amplicon PCR Forward = 5′ TCGTCGGCAGCGTCAGATGTGTATAAGAGACAGCCTACGGGNGGCWGCAG and Reverse = 5′ GTCTCGTGGGCTCGGAGATGTGTATAAGAGACAGGACTACHVGGGTATCTAATCC primers following the PCR protocol suggested by Illumina.

PCR products were purified using the Agentcourt^®^ AMPure^®^ XP Beads (Beckman Coulter Company, Brea, CA, United States). The quality of the final products was assessed using a Bioanalyzer 2100 (Agilent Technologies, Waldbronn, Germany) and quantified with Qubit^®^ fluorometer (Thermo Fisher Scientific, Waltham, MA, United States) following manufacturer protocol. The amplicons were coupled to dual indices and Illumina sequencing adaptors attaches using the Nextera XT Index Kit (Illumina Inc., San Diego, CA, United States), pooled in equal proportions and sequenced paired-end in an Illumina MiSeq (Illumina Inc., San Diego, CA, United States) at IGA Technology Services (Udine, Italy). To prevent focusing and phasing problems due to the sequencing of “low diversity” libraries such as 16S amplicons, 30% PhiX genome was spiked in the pooled library.

### Bioinformatic Analysis

Raw reads were first processed with Trimmomatic (Bolger et al., 2014) to remove low-quality reads using a sliding window of 5 bp length with an average phred score ≥ 20. Sequences shorter than 100 bases were discarded. The 16S rRNA sequences were analysed using the Mothur software package version 1.35.1 (Schloss et al., 2009). The paired-end reads were assembled and aligned to the SILVA 16S rRNA sequences database (Pruesse et al., 2007). Sequences were de-noised to remove sequencing error with the command “pre.cluster” and chimeric sequences were removed using the Uchime algorithm (Edgar et al., 2011) implemented in Mothur. Sequences were clustered into OTUs at 96% sequence identity using the nearest neighbour clustering methods. The sequences were classified using the references Ribosomal Database Project database (RDP) provided in Mothur. OTUs that were singletons and doubletons were removed. The samples were normalised to 6,886 sequences each (the size of the smallest sample) to ensure that the analysis was not influenced by differential sequencing depths. The bacterial 16S rRNA gene Illumina sequencing data are deposited in the NCBI BioProject library (Accession: PRJNA472855, ID: 472855). The bacterial taxonomic table (with bacterial relative abundance data) are given in Supplementary Table S1.

### qPCR Analysis

The primer sets used in this study are listed in Table 1. New primer sets were designed based on *L. plantarum* WCFS1 and *P. syringae* pv. *actinidiae* RC3 sequences available at the National Centre for Biotechnology Information (NCBI^1^). Appropriate primers were designed using the online programme Primer3Plus^2^ (Untergasser et al., 2012). The BLAST search software (Basic Alignment Search Tool^3^) was used to cheque the specificity of each primer set. Properties of each primer were verified by Oligo analyser 3.1. Primer specificity was validated by melt curve analysis and end point PCR performed with the same protocol adopted for qPCR (see below), using AmpliTaq Gold^®^ 360 enzyme and Master Mix (Thermo Fisher Scientific, Waltham, MA, United States).

**Table 1:** Primers used in this study to reveal the presence of the indicated bacterial species.

Species	Forward	Reverse	Reference
*P. syringae* pv. *actinidiae*	ACACCGCCCGTCACACCA	GTTCCCCTACGGCTCCT	This study
*P. syringae* pv. *syringae*	TCCTTATCGATCTGCAACTGGCGA	ATGGTTGCCTGCAGTTCATTCCC	Najafi Pour and Taghavi, 2011
*P. viridiflava*	GTAGGTGGTTTGTTAAGTTGAA	GTAGGTGGTTTGTTAAGTTGAA	Alimi et al., 2011
*P. fluorescens*	TGCATTCAAAACTGACTG	AATCACACCGTGGTAACCG	Scarpellini et al., 2004
*L. plantarum*	TTTGAGTGAGTGGCGAACTG	CGAAGCCATCTTTCAAGCTC	This study
*B. subtilis*	AATGACCGTGCTCCATCTGTAA	TTCCGATCTTTAACGGATTGCT	Rotolo et al., 2016
*B. amyloliquefaciens*	CCGGCGAAATCAAAATAATGAC	GGCAGGATCATACGGGAGAA	Rotolo et al., 2016
*P. agglomerans*	ACGGTGCGTTCCGCAATA	GGCGCCGGGAAAACATAC	Braun-Kiewnick et al., 2012

qPCR analyses were performed using Sybr Green fast master mix chemistry (Applied Biosystem, Foster City, CA, United States) in a 96 well spectrofluorometric thermal cycler StepOnePlus^®^ (Thermo Fisher Scientific, Waltham, MA, United States). DNA concentration was adjusted to 100 ng. All reactions were performed in triplicate, with the following thermal profile: 1 cycle at 50°C (2 min), 1 cycle at 95°C (10 min), 40 cycles of 95°C (15 s) and 60°C (30 s). The temperature was raised by 0.3°C every 10 s from 63 to 95°C to obtain the melting temperature. To quantify the bacterial titre of the samples, standard curves were generated for each bacterial species tested plotting cycle threshold (Ct) values versus bacterial cell titre, as measured by plating 10-fold dilutions of the same sample on LB-agar medium (Lyons et al., 2000). Upon verification by genome blast, the average number of detector gene copies per genome was assumed to be 1.0 for each species.

### Meta-Analysis of Bacterial Association

The correlation among natural epiphytic populations of Psa and other bacteria (*P. syringae* pv. *syringae, P. viridiflava, P. fluorescens, Pantoea agglomerans*/*vagans*, and *Lactobacillus* spp.) was evaluated based on data obtained between 2012 and 2016, relating to *A. deliciosa* cv. Hayward samples collected in the same area and season as the samplings for metagenomic analysis. Each sample was singularly washed in 10 ml MgSO_4_ 10 mM sterile solution. Bacterial quantification was performed on the wash by qPCR as described above.

### Statistical Analysis

To assess the coverage of the sequencing depth, individual rarefaction analysis was performed for each sample using the “diversity” function in PAST 3.0 (Hammer et al., 2001). Alpha diversity indices (Pielou, Inverse Simpson and Shannon) were analysed after normalisation using Mothur. Similarity Percentages (SIMPER) analysis using PAST was used to calculate the average dissimilarity and to obtain the identity and relative abundances of the bacterial taxa that contributed most of the observed pair-wise variation in the bacterial community composition due to different kiwifruit species (healthy plants), organs (healthy plants) and pathological status (healthy vs. diseased plants). Principal component analysis (PCA) based on correlation matrix was carried out in PAST to display the clusterisation of samples according to the variance in qPCR population analysis.

Multiple regression was performed on bacterial populations to test their association with Psa, using Statistica ver. 7.0 (Statsoft, Inc., Tulsa, OK, United States). The analysis was restricted to samples positive to Psa, and the data were transformed to Log_10_ before elaboration. Statistical significance was assumed for *P* < 0.05.

## Results

### Description of the Epiphytic Bacterial Microbiome of Kiwifruit Plant

**FIGURE 1:**
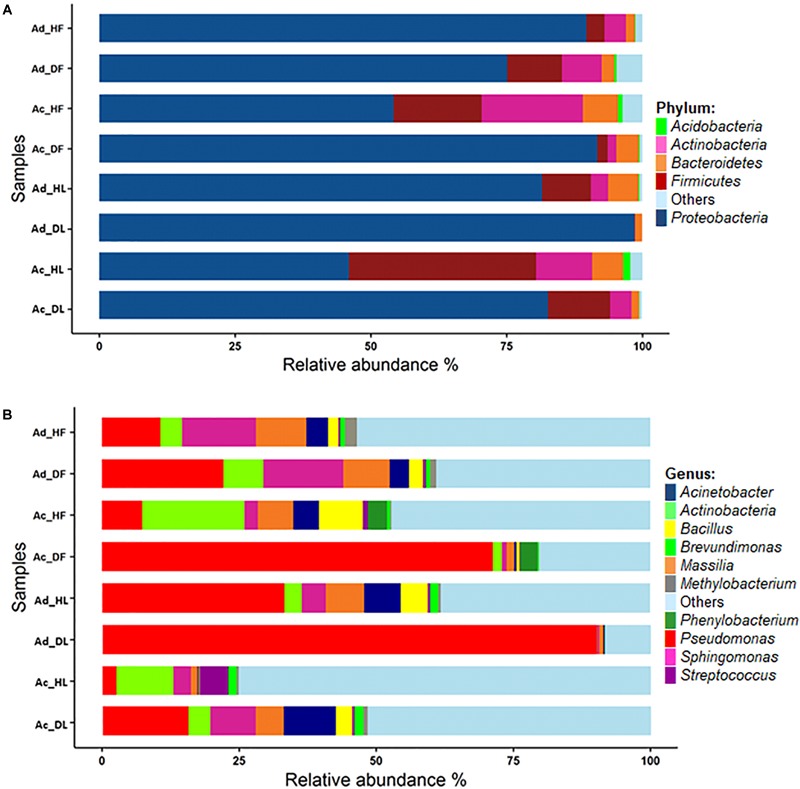
Bacterial community composition at the phylum **(A)** and genus level **(B)**. In the stacked bar plot are reported the most abundant phyla and genera. In the sample codes, Ac, *Actinidia chinensis* var. HORT16A; Ad, *Actinidia deliciosa* var. Hayward; D, diseased; H, healthy; F, flower; L, leaf.

In this study, the leaf- and flower-associated microbiota of two kiwifruit species, *A. deliciosa* and *A. chinensis*, were analysed. After the normalisation step at 6,886 sequences per sample, a total of 1,050 bacterial OTUs were retrieved. The rarefaction curves were close to saturation, suggesting that the OTUs recovered in this study nearly represented the whole bacterial genetic diversity (Supplementary Figure S1). The OTUs were assigned to 16 phyla, and 220 different genera (Supplementary Table S1). *Proteobacteria* was the most abundant phylum representing about 77.4% of the total contigs, followed by *Firmicutes* (10.7%), *Actinobacteria* (6.1%), and *Bacteroidetes* (3.5%) (Figure 1). At an OTU level, the most abundant OTUs were identified as *Pseudomonas* OTU 00001 (31.5%), two unclassified genera from the *Enterobacteriaceae* family, OTU 00002 and OTU 00004, representing together about 15.3% of the total sequences, *Sphingomonas* OTU 00003 (5.8%) and *Massilia* OTU 00005 (4.9%). The first 10 most abundant OTUs accounted for approximately 70% of total sequences (Figure 1 and Supplementary Table S1).

### Species-Specificity of Epiphytic Bacterial Microbiome

**FIGURE 2:**
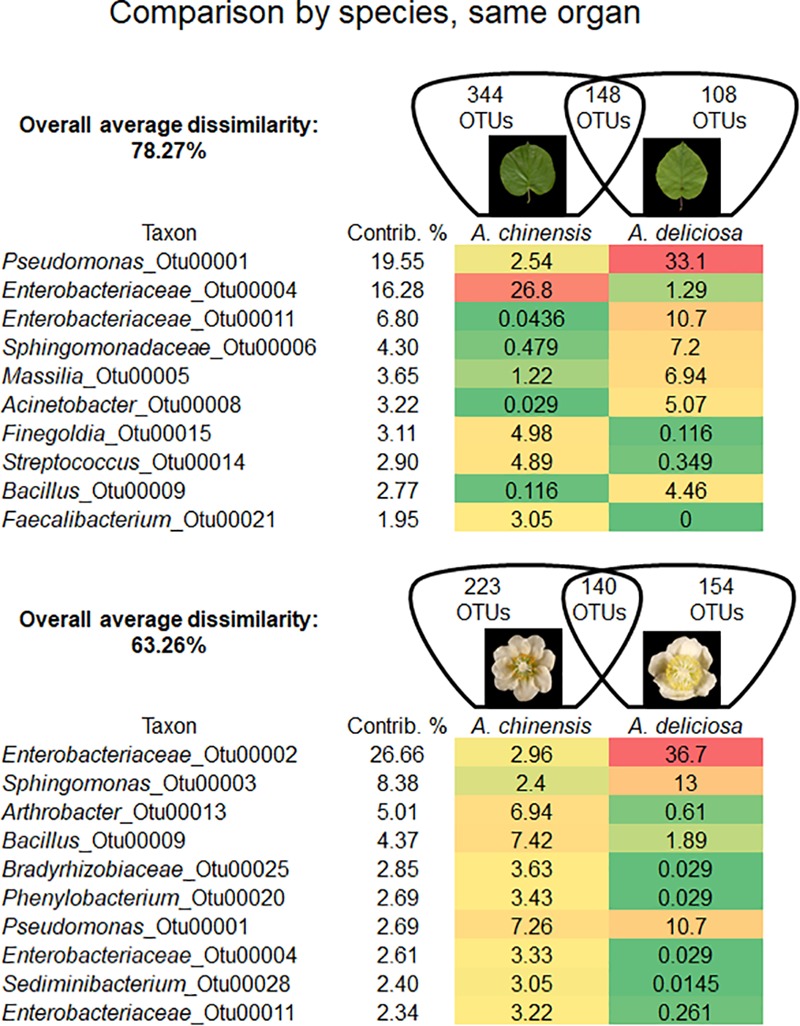
Overall average dissimilarity between the same organs, leaves and flowers, of two kiwifruit species, *A. chinensis* and *A. deliciosa*. For each organ, the numbers of OTUs specific to one of the two species, or present in both of them, are indicated in the Venn diagrams. Top ten OTUs which contribute most to the overall average dissimilarity and their relative abundances are shown. The colour scale indicates the abundance ranking of the relative OTU: highest (red), mid-point with 50% percentile (yellow), lowest (green).

Bacterial community was shaped by the species of kiwifruit plants (Figure 2). In fact, the overall average dissimilarity between leaves or flowers of the two species was 78.27 and 63.26%, respectively. In leaves, two unclassified genera (OTU 00004 and 00011) belonging to *Enterobacteriaceae* accounted for about 23% of the dissimilarity. The genus *Pseudomonas* (OTU 00001) also accounted approximately for 20% of the dissimilarity, being 13 times more abundant on *A. deliciosa* than on *A. chinensis* leaves.

On flowers, *Enterobacteriaceae* (OTU 00002) accounted for approximately 27% of dissimilarity followed by the genus *Sphingomonas* (8.4%, OTU 00003). The genera *Arthrobacter* (OTU 00013) and *Bacillus* (OTU 00009) accounted for 5 and 4% of dissimilarity, respectively. *Pseudomonas* (OTU 00001) accounted only for the 2.7% of dissimilarity.

The influence of kiwifruit plant species was confirmed also by the three biodiversity indices determined: Shannon (*H*′), Inverse Simpson (1/*D*′) and Pielou (*J*′). A higher biodiversity was observed in *A. chinensis* leaves (*H*′ = 4.02; 1/*D*′ = 11.93; *J*′ = 0.65) than in *A. deliciosa* ones (*H*′ = 2.99; 1/*D*′ = 7.16; *J*′ = 0.54). Similarly, higher values of all the considered indices were found for *A. chinensis* (*H*′ = 4.13; 1/*D*′ = 30.14; *J*′ = 0.70) than for *A. deliciosa* (*H*′ = 2.76; 1/*D*′ = 5.73; *J*′ = 0.49) flowers (Table 2).

**Table 2:** Summary of diversity estimate for each sample.

Sample	Total OTUs	Shannon index (*H*′)	Simpson reciprocal index (1/*D*′)	Pielou index (*J*′)
Ac_DF	173	1.61	1.96	0.31
Ac_HF	363	4.13	30.14	0.70
Ad_DF	411	3.49	10.84	0.58
Ad_HF	294	2.76	5.73	0.49
Ac_DL	246	2.95	8.09	0.54
Ac_HL	492	4.02	11.93	0.65
Ad_DL	59	0.57	1.23	0.14
Ad_HL	256	2.99	7.16	0.54

*Ac, Actinidia chinensis var. HORT16A; Ad, Actinidia deliciosa var. Hayward; D, diseased; H, healthy; F, flower; L, leaf. For each sample, 6,886 sequences were analysed*.

### Organ-Specific Epiphytic Bacterial Microbiome

**FIGURE 3:**
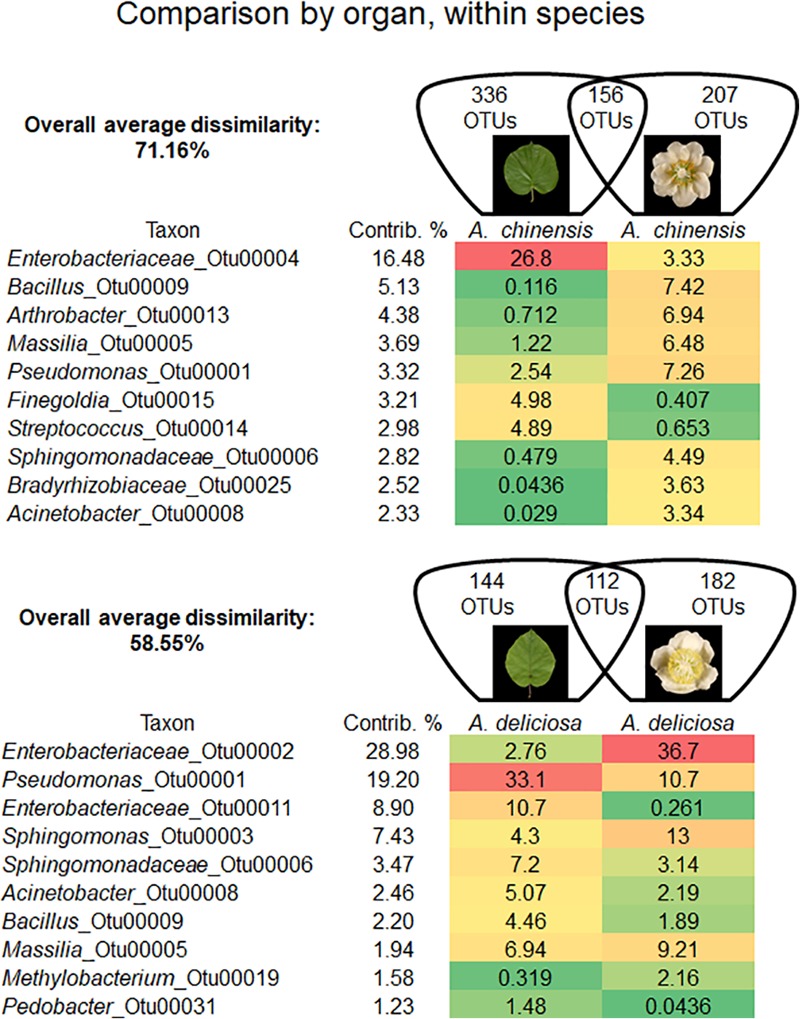
Overall average dissimilarity between leaves and flower of the same kiwifruit species, *A. chinensis* or *A. deliciosa*. For each species, the numbers of OTUs specific to one of the two organs, or present in both of them, are indicated in the Venn diagrams. Top ten OTUs which contribute most to the overall average dissimilarity and their relative abundances are shown. The colour scale indicates the abundance ranking of the relative OTU: highest (red), mid-point with 50% percentile (yellow), lowest (green).

In each kiwifruit species, flowers and leaves harboured a distinct bacterial microbiome. In fact, in *A. chinensis* the overall average dissimilarity between leaves and flowers was 71.16%, while for *A. deliciosa* it was 58.55% (Figure 3). In *A. chinensis*, an unclassified genus (OTU 00004) belonging to *Enterobacteriaceae* accounted for 16.48% of dissimilarity followed by the genus *Bacillus* (OTU 00009, 5.13%). On the other hand, in *A. deliciosa* OTU 00002 belonging to *Enterobacteriaceae* accounted for about 29% of the dissimilarity being 13 times more abundant on flowers, while the genus *Pseudomonas* (OTU 00001) accounted for approximately 19% of dissimilarity and it was more abundant on leaves than flowers (Figure 3).

These data are in agreement with the bioversity indices trend. *A. chinensis* hosted a more biodiverse epiphytic bacterial community (*H*′ = 4.13; 1/*D*′ = 30.14; *J*′ = 0.70) than leaves (*H*′ = 4.02; 1/*D*′ = 11.93; *J*′ = 0.65). On the other hand, in *A. deliciosa*, the bacterial community presented a higher complexity in leaves (*H*′ = 2.99; 1/*D*′ = 7.16; *J*′ = 0.54) than flowers (*H*′ = 2.76; 1/*D*′ = 5.73; *J*′ = 0.49) (Table 2).

### Effect of Psa Infection on the Epiphytic Bacterial Microbiome

**FIGURE 4:**
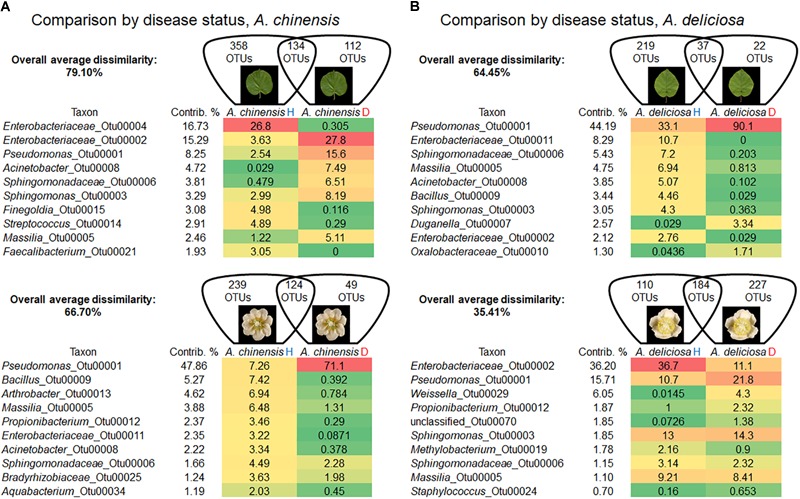
Overall average dissimilarity between leaves and flower of healthy and infected *A. chinensis* plants **(A)** and *A. deliciosa* **(B)** (H, healthy; D, diseased, infected with *Pseudomonas syringae* pv. *actinidiae*). For each combination of species and organ, the numbers of OTUs specific to H or D samples, or present in both of them, are indicated in the Venn diagrams. Top ten OTUs which contribute most to the overall average dissimilarity and their relative abundances are shown. The colour scale indicates the abundance ranking of the relative OTU: highest (red), mid-point with 50% percentile (yellow), lowest (green).

A detailed comparison about bacterial community changes related to Psa infection showed that the overall average dissimilarity between leaves and flower of healthy and infected plants ranged from 79.10 to 66.70% in *A. chinensis* and from 64.45 to 35.41% in *A. deliciosa* (Figures 4A,B).

In *A. chinensis*, the substantial increase in the genus *Pseudomonas* (OTU 00001) in infected leaves and flowers contributed 8.25 and 47.86%, respectively (Figure 4A). A similar result was observed in *A. deliciosa* were the genus *Pseudomonas* (OTU 00001) increased up to three and two times in leaves and flowers, respectively (Figure 4B).

A reduction in diversity of the bacterial community was observed after Psa infection, with the only exception of *A. deliciosa* flowers, as indicated by the biodiversity indices (Table 2). Psa infection caused a marked drop in population evenness and biodiversity in infected *A. chinensis* (flowers and leaves) and *A. deliciosa* (leaves only), with the dominance of few genera, mainly *Pseudomonas* (OTU 00001).

**FIGURE 5:**
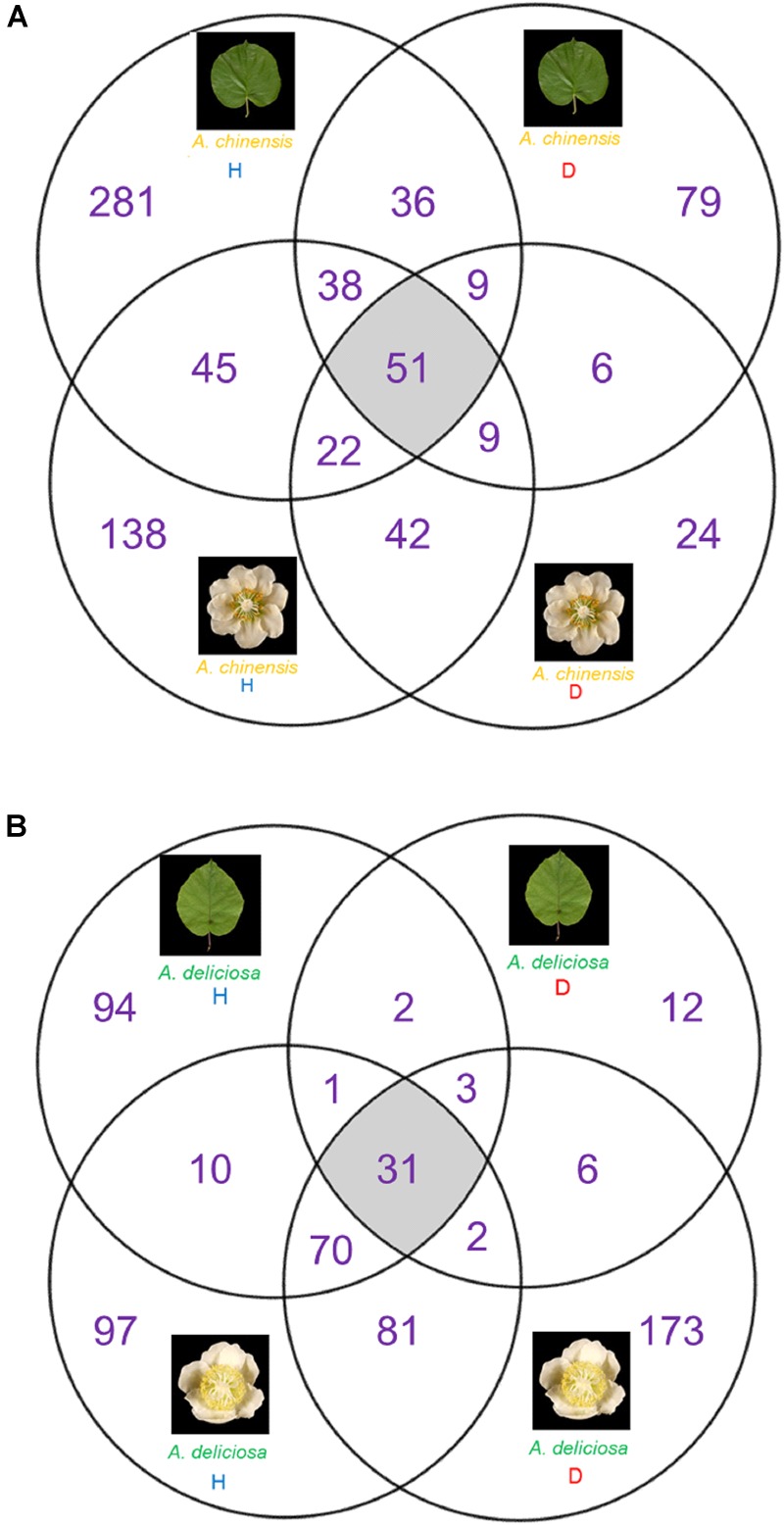
Venn diagrams showing the unique and shared OTUs between healthy and diseased tissue. **(A)** *A. chinensis* diseased and healthy leaves and flowers. **(B)** *A. deliciosa* diseased and healthy leaves and flowers.

The Venn diagrams in Figure 5 describe the distributions of unique and shared OTUs in healthy and diseased plants in the two species and tissues analysed. Psa infection had the strongest impact on the bacterial community of *A. deliciosa* leaves compared to the other conditions analysed, only 37 OTUs being shared between healthy and diseased leaves. Furthermore, on infected leaves of *A. deliciosa* we identified some specific OTUs belonging to *Oxalobacteraceae* (OTU 00043, OTU 00186), *Haemophilus* (OTU 00076), *Moraxellaceae* (OTU 00323) that were not present in healthy *A. deliciosa* leaves (Supplementary Table S1). Finally, a considerable number of high abundance OTUs disappeared or showed a dramatic reduction on leaves from infected plants (Supplementary Table S1). A similar trend was observed in *A. chinensis* flowers and leaves. Also in this case, healthy plants were characterised by the presence of characteristic OTUs absent in diseased ones (e.g., *Epilithonimonas* OTU 00016, *Porphyromonas* OTU 00110, *Bacteroides* OTU 00111).

On the other hand, in *A. deliciosa* flowers some OTUs were more abundant or only present in diseased samples (e.g., *Pseudomonas* OTU 00001, *Propionibacterium* OTU 00012, *Weissella* OTU 00029) (Supplementary Table S1).

### Abundance and Dynamic of *Pseudomonas* spp. Pathogens and Putative Biocontrol Bacterial Agents in Relation to Plant Pathological Status

Quantitative PCR was applied to detect the occurrence, in relation to plant pathological status, of two other kiwifruit pathogenic bacteria: *P. syringae* pv. *syringae* (Pss) and *P. viridiflava* (Pv) and bacterial species with potential biocontrol activity, such as *P. agglomerans/vagans, L. plantarum, B. subtilis, B. amyloliquefaciens*, and *P. fluorescens* (Choudhary and Johri, 2009; Savitha et al., 2013; Bonaterra et al., 2014; Dutkiewicz et al., 2016; Sharifazizi et al., 2017). Novel primer sets were developed for *L. plantarum* and Psa. Melting curves analysis (Supplementary Figure S1) revealed the specificity of designed primers: a unique peak was observed, suggesting the specificity of the amplification, i.e., each primer pair amplified a unique locus targeted on the genome.

**FIGURE 6:**
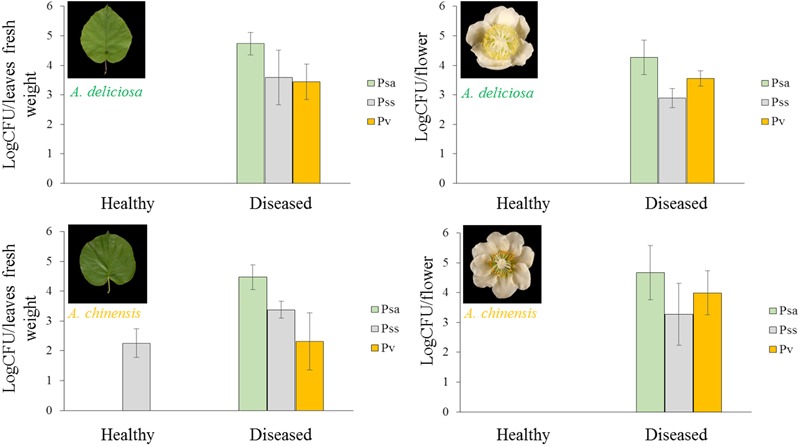
Abundance in each sample (mean ± standard error, *n* = 5) of *Pseudomonas syringae* pv. *actinidiae* (Psa), *Pseudomonas syringae* pv. *syringae* (Pss), and *Pseudomonas viridiflava* (Pv) determined by qPCR.

**FIGURE 7:**
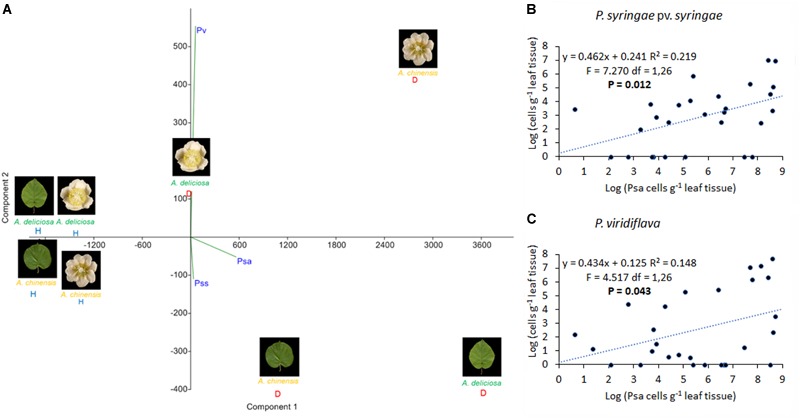
Association of *Pseudomonas syringae* pv. *actinidiae* (Psa) with *P. syringae* pv. *syringae* (Pss) and *P. viridiflava* (Pv). **(A)** PCA ordination (with biplot function) showing the distribution of samples from healthy and infected plants on the basis of pathogens quantification by qPCR. **(B)** Linear regression between Psa and Pss populations. **(C)** Linear regression between Psa and Pv populations in infected flowers. In **(B,C)**, the data sets were restricted to samples presenting a Psa population lower than 10^9^ bacterial cells flower^-1^.

In Psa-infected plants, all the three pathogens were present, although Pss and Pv populations were generally lower than Psa (Figure 6). Psa and Pss/Pv were associated in 62.5% of flowers. In non-infected samples, none of the pathogens was detected, with the only exception of a small amount of Pss on the leaves of *A. chinensis* (Figure 6). PCA analysis revealed that healthy plants clustered together. In particular, diseased flowers of both species were mainly characterised by presence of Pv, while Psa and Pss were mainly associated to diseased leaves (Figure 7). A significant correlation was found between epiphytic Psa and Pss/Pv populations, for values lower than 10^9^ bacterial cells per flower (Figures 7B,C).

**FIGURE 8:**
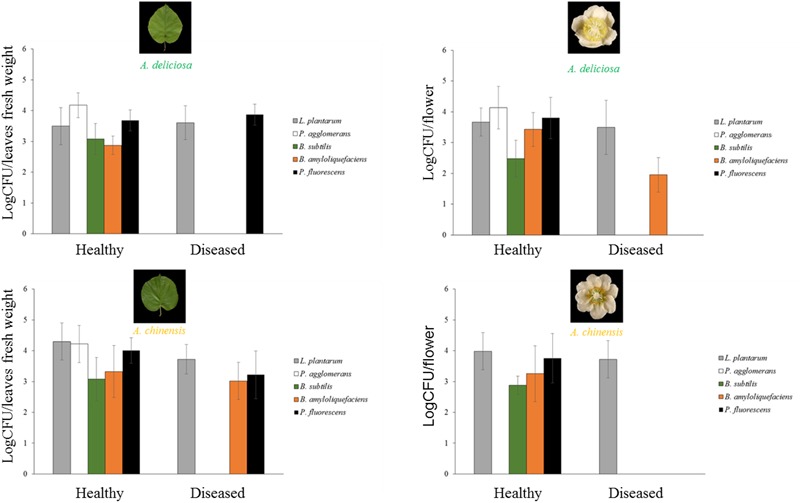
Abundances (mean ± standard error, *n* = 5) of *L. plantarum, P. agglomerans, B. subtilis, B. amyloliquefaciens*, and *P. fluorescens* determined by qPCR.

**FIGURE 9:**
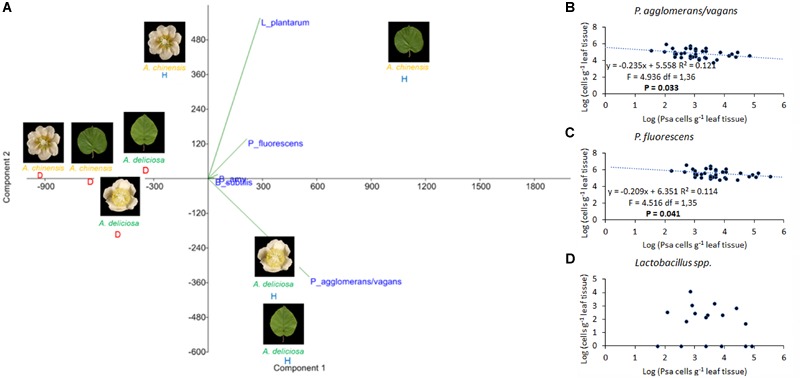
Association of *Pseudomonas syringae* pv. *actinidiae* (Psa) with *Pantoea agglomerans*/*vagans, P. fluorescens, Lactobacillus plantarum*, and Bacillus spp. **(A)** PCA ordination (with biplot function) showing the distribution of samples from healthy and infected plants on the basis of BCAs quantification. **(B)** Linear regression between Psa and *Pantoea agglomerans*/*vagans* populations. **(C)** Linear regression between Psa and *P. fluorescens* populations. **(D)** Scatterplot representing Psa and *Lactobacillus* spp. populations on leaf samples. In **(B–D)**, the data sets were restricted to samples presenting a Psa population lower than 10^6^ bacterial cells g^-1^ tissue.

Regarding the bacterial species with potential biocontrol activity (*P. agglomerans/vagans, L. plantarum, B. subtilis, B. amyloliquefaciens*, and *P. fluorescens*), all of them were found in the healthy plant samples, while only *L. plantarum* appeared also in the corresponding infected organs, and *P. fluorescens* was present in diseased leaves, but not flowers (Figure 8). *B. amyloliquefaciens* appeared in some diseased samples, without connection to the plant species or organ. The other species were not present in diseased samples. *P. agglomerans*/*vagans* and *P. fluorescens* population sizes were inversely correlated with Psa, for pathogen populations lower than 10^5^ and 10^6^ bacterial cells per gramme of tissue, respectively. No significant correlation was found with *Lactobacillus* spp. Principal component analysis showed that diseased plants were well differentiated from healthy ones and that the presences of *L. plantarum* and *P. agglomerans/vagans* were mostly distinctive of *A. chinensis* and *A. deliciosa*, respectively (Figure 9).

## Discussion

### Microbial Biodiversity in *Actinidia* Phyllosphere

The phyllosphere supports complex microbial populations, and the phyllosphere microbiota can promote plant growth or exhibit biocontrol against various plant pathogens (Thapa et al., 2017). The abundance and spatial distribution of phyllosphere microbiota is to a large extent influenced by environmental factors, but host plant genotype also plays a key role (Bringel and Couée, 2015). Indeed, in previous research, a core community of 31 bacterial species, amounting to 99.8% of total sequences, was found on kiwifruit pollen samples regardless of the different geographical origins and year of collection (Kim M.J. et al., 2018). The present study provides a comprehensive description of the epiphytic bacterial communities on flowers and leaves of *A*. *chinensis* and *A. deliciosa*, the two main kiwifruit commercial species, and highlights their variability in relation to Psa infection. The differences in the microbiota structures were investigated also through the determination of three biodiversity indices. Shannon and Inverse Simpson indices were used to extrapolate the total richness from the observed OTUs. The former is the most widely used index based on species richness and is sensitive to changes in rare species, while the latter is preferred over other measures of alpha-diversity because it accounts for evenness in addition to the number of species. Finally, Pielou index provides information on species evenness, ranging from 0 to 1, with 1 representing perfect evenness and 0 complete dominance. In kiwifruit phyllosphere, *Proteobacteria, Firmicutes, Actinobacteria*, and *Bacteroidetes* were the most abundant phyla. These phyla are considered as phyllosphere-associated generalists and have been found to be the most abundant phyla in the phyllosphere of several plant species (Bulgarelli et al., 2013; Bringel and Couée, 2015; Kim M.J. et al., 2018). The most frequent genera were *Pseudomonas, Sphingomonas*, and *Massilia*. Their presence has been reported also by other authors in other host plants (López-Velasco et al., 2011; Bodenhause et al., 2013; Bogas et al., 2015). The predominant genus was *Pseudomonas*. In general, pseudomonads colonise plant surfaces, and many strains harbour interesting potential biocontrol actions (Thapa et al., 2017). Some species, such as *P. putida*, are known for their phosphate solubilisation ability and IAA production (Adesemoye et al., 2008; Gholami et al., 2009; Ahemad and Khan, 2012; Ahemad and Kibret, 2014). Strains of *P. fluorescens, P. aeruginosa, P. asplenii*, and *P. protegens* are also used as biocontrol agents against different pathogens (Krishnamurthy and Gnanamanickam, 1998; Akter et al., 2016; Michavila et al., 2017).

The *Actinobacteria* class also represents a reservoir of potential BCAs. Members of this phylum are well known for their ability to produce secondary metabolites with application in the agricultural, pharmaceutical and medical industries (Himaman et al., 2016). Several studies proposed their use as BCAs (El-Tarabily et al., 2000; Kunoh, 2002; Cao et al., 2005; Prapagdee et al., 2008; Mingma et al., 2014). They play key roles as plant growth promoters, disease resistance inducers and drought tolerance stimulators (Himaman et al., 2016).

The genus *Sphingomonas* generally acts as a plant-protective genus by suppressing disease symptoms and decreasing pathogen growth (Kim D. et al., 2018). Innerebner et al. (2011) showed that the inoculation with a *Sphingomonas* sp. strain reduced the population size of the plant pathogens *Pseudomonas syringae* pv. *tomato* DC3000 and *Xanthomonas campestris* pv. *campestris* LMG 568 on *Arabidopsis* leaves. The genus *Massilia* belongs to the family of *Oxalobacteraceae*. The presence of *Massilia* spp. was reported in the phyllosphere of different plants, including lettuce and apple (Bassas-Galia et al., 2012; Rastogi et al., 2012; Yashiro and McManus, 2012).

### Host-Specific Bacterial Communities

Both on flowers and on leaves, the epiphytic bacterial community differed according to kiwifruit species. The main taxa contributing to differences were *Enterobacteriaceae, Pseudomonas, Acinetobacter*, and *Sphingomonadaceae* on leaves, and *Sphingomonas, Arthrobacter, Bacillus*, and *Bradyrhizobiaceae* in flowers. The detection of this last genus has been reported also in the phyllosphere of spinach, rice, and tobacco, providing evidence for vertical transmission of bacteria from seed to the phyllosphere (Chi et al., 2005; Li et al., 2010; Lopez-Velasco et al., 2013).

Data obtained here are in agreement with other studies showing that different plants genotypes of the same species can host different bacterial communities (De Costa et al., 2006; Vorholt, 2012). The observed differences could be related to anatomical differences of leaves and flowers of the two kiwifruit species. In fact, bacteria are not uniformly distributed across leaf surfaces: instead, they form scattered microcolonies in proximity of trichomes, stomata, epidermal cell wall junctions and grooves along veins (Lindow and Brandl, 2003; Vorholt, 2012), where water and nutrients are most available (Kinkel, 1997; Leveau and Lindow, 2001; Monier and Lindow, 2004; Vorholt, 2012). One of the most considerable anatomical differences between *A. deliciosa* and *A. chinensis* is related with trichome structure and density. In *A. chinensis*, the leaves show a higher trichome density compared to *A. deliciosa* cultivars (He et al., 2000; Spinelli et al., 2011). Moreover, trichomes in *A. chinensis* are characterised by a higher central peduncle (He et al., 2000). These differences in trichomes abundance may affect the microbial community composition and structure.

### Organ-Specific Bacterial Communities

Differences in the microbial community were also observed in different organs of the same plant species. *A. chinensis* leaves were populated mostly by *Enterobacteriaceae* and *Pseudomonas*, while on flowers, *Pseudomonas* and *Bacillus* were the most abundant genera, and a higher overall biodiversity was observed. Contrastingly, in *A. deliciosa*, the influence of organs on microbial composition was less evident than in *A. chinensis*, as *Pseudomonas* and *Enterobacteriaceae* were the dominant groups on both leaves and flowers. Organ-specific pathogenic consortia were observed in Psa-infected host. In fact, Pss, which primarily induces leaf spot symptoms (Petriccione et al., 2017), was more abundantly found on Psa-infected leaves, while Pv, responsible for blossom blight disease (Balestra et al., 2008) was more closely associated to Psa-infected flowers.

Since flowers are composed by different tissues (stigmas, styles, anthers, ovariums, nectarhodes), each of them providing a favourable and unique environment for the resident microbial community (Howpage et al., 1998; Spinelli et al., 2005; Aleklett et al., 2014), a higher biodiversity on flowers than on leaves could be expected. However, some flower parts may be less conducive to bacterial epiphytes than leaves. In fact, Junker et al. (2011) observed a lower biodiversity on petals than leaves of *Lotus corniculatus* and *Saponaria officinalis*. In this perspective, the relative organisation, proportion, and chemical features of *Actinidia* spp. flower parts may be evoked to explain differences in bacterial colonisation. Although sharing the same basic structure, *A. chinensis* and *A. deliciosa* flowers also show evident morphological differences. *A. deliciosa* flowers present a higher number of styles and stamens, and the perianth and androecium are closer to gynoecium, resulting in the nectar cup being more protected than in *A*. *chinensis* (Harvey and Fraser, 1988; Huang, 2014). It is likely that this last evidence could pose an obstacle to the formation of a highly diversified community, as observed on *A. deliciosa* flowers compared to leaves. Moreover, the production of volatile compounds may further act as a selective agent on the epiphytic microflora (Junker et al., 2011). The sesquiterpene α-farnesene, for instance, was found to play a role in plant defence (Huelin and Murray, 1966; Pare and Tumlinson, 1999; Yang et al., 2011), acting as a feeding deterrent to insects (Aharoni et al., 2003) and exhibiting toxicity to bacteria (Chorianopoulos et al., 2004) and fungi (Terzi et al., 2007). This compound is a major constituent of the flower odour bouquet of *A*. *deliciosa*, while it is not or scarcely emitted by *A. chinensis* (Tatsuka et al., 1990; Crowhurst et al., 2008; Nieuwenhuizen et al., 2009; Green et al., 2012).

### Antagonistic and Synergic Relationships of Psa With Other Microbes

*Pseudomonas syringae* pv. *actinidiae* infection had several effects on the diversity and taxonomic structure of the bacterial communities with the only exception of *A. deliciosa* flowers. The mechanism by which Psa antagonises indigenous bacteria, determining its dominance on *A. chinensis* and *A. deliciosa* leaves and the disappearance of most of the dominant microbial species, is probably based on competition for limiting nutrient resources, since a mechanism based on antibiosis would not be affected by the plant host. In this view, a specialised pathogen such as Psa should be able to outcompete, in the *Actinidia* phyllosphere, other non-specialised residents. The extensive screening of plant material collected from infected orchards for 4 years confirmed a negative correlation between Psa and *P. agglomerans*/*vagans* or *P. fluorescens* populations when the population of these two bacteria ranges between 10^4^ and 10^6^. Thus, even though in highly infected leaves, Psa overwhelms other bacterial competitors, in early stages of Psa epiphytic growth the competition with *P. agglomerans*/*vagans* or *P. fluorescens* may prevent the reach of the infection threshold (approximately 10^5^ Psa cells per gramme of tissue) (Donati et al., 2018). On the other hand, no antagonism could be observed between Psa and *Lactobacillus* spp., speculatively suggesting that bacteria of the latter group are poorly affected by Psa competition. In comparison to leaves or *A. chinensis* flowers, Psa exerts a weak antagonism toward the other residents in *A. deliciosa* flowers (Figure 1B and Table 2). Thus, it may be concluded that this niche is not highly conducive for Psa, or that other bacteria are as specialised and adapted to the floral niche as Psa. This observation, together with the higher relative abundance of Psa on *A. chinensis* flowers, could also contribute in explaining the higher susceptibility of these flowers in comparison with *A. deliciosa* ones (Donati et al., 2018), in spite of the similar Psa population sizes that can be attained on the two species (Figure 6).

Quantitative real-time polymerase chain reaction analysis showed that Psa formed a syndemic association with Pss and Pv. In fact, Psa-infected flowers also harboured detectable Pss and Pv populations in 62.5% of the cases. It is reasonable to hypothesise that these three species form a consortium which may compete more effectively in the epiphytic niche (Buonaurio et al., 2015). When occurring in association, Psa and Pss have been shown to infect the host plant more efficiently (Petriccione et al., 2017). Such observations pose interesting implications for the control of bacterial canker of kiwifruit. For instance, treatments aimed at reducing Pss and/or Pv population may be envisaged to limit Psa pathogenicity. In addition, hacking the signalling network among different pathogen species may be a strategy to repress their virulence in field conditions. Many Gramme-negative, plant-associated bacteria pathogens have been reported to regulate their virulence by *N*-acyl-homoserine lactones (AHLs) (Ma et al., 2013). AHL-quorum sensing model includes AHL synthase, which belongs to the LuxI-protein family, and AHL receptors/transcriptional regulators (LuxR) (Papenfort and Bassler, 2016). Psa does not produce AHLs and a complete LuxI/R system is absent. However, it possesses three putative LuxR solos (Patel et al., 2014). Two of them respond to exogenous AHLs, while the third is most likely involved in interkingdom signalling (Patel et al., 2014). The presence of three LuxR solos is rather unusual as most commonly proteobacteria possess only one, therefore they could represent an evolutionary advantage for Psa favouring the communication with other epiphytic bacteria (Patel et al., 2014).

Finally, inside this bacterial consortium, horizontal gene transfer may be facilitated, thus allowing a faster adaptation to environmental changes and stresses. In fact, the accessory genome of the *P. syringae* complex is characterised by genomic islands and various mobile elements, such as insertion sequences (IS elements), transposons, plasmids and integrative conjugative elements (ICEs) (Butler et al., 2013). These mobile elements include genes related to the ecological fitness and virulence, such as toxin production (Murillo et al., 2011), copper and antibiotic resistance (Butler et al., 2013; Colombi et al., 2017), siderophore production and phenolics degradation (Scortichini et al., 2012). In this sense, the possibility to characterise the “phyllobiome” through the application of high throughput, next generation sequencing technologies allows to strategically select BCAs highly specialised for the host’s most sensible pathways of infection. Among the putative BCAs studied in this work, for instance, only *L. plantarum* colonised both infected and healthy flowers and leaves in the two *Actinidia* species, while *B. amyloliquefaciens* could be occasionally found in infected tissues, although without relation to plant organ or species. Furthermore, coupling the complementary information acquired by next generation sequencing technologies and metanalysis of population association, allowed to identify other BCA candidates, such as *P. agglomerans*/*vagans* or *P. fluorescens.* The ability of tested BCAs to persist in these organs in spite of Psa infection could be related with the ability of members of this species to exert a strong antagonistic effect against the other microbes inhabiting the same niche thorough the release of a wide array of anti-microbial compounds opening new possibilities for its exploitation as BCAs.

## Conclusion

The data obtained in this work highlighted for the first time the impact of Psa on bacterial communities associated to kiwifruit plants. The results here reported provide new insight on how Psa influences the microbial communities associated with the leaf and the flower in kiwifruit. The complex interactions between host, environment, and microbes play a role in determine the outcome of the infection process and in defining niches important for the resident bacteria. The main causes underlying these changes remain unclear, and a better understanding of the process will require a greater interdisciplinary effort, as well as an integrative approach to detect the triggers of disease outbreak in kiwifruits, to develop more sustainable strategies for the control of the bacterial canker disease in kiwifruit.

## Author Contributions

FS conceived the experiment and supervised the work. ID contributed to design of the experiments and performed all the sampling and the classical microbiological and pathological analysis. AC and ID performed DNA extraction, qPCR and bacterial identification. LO, AL, and WP analysed the next generation sequencing data. FS, LO, ID, and GP drafted the manuscript. All authors critically contributed to the review of the manuscript and discussion of the data.

## Conflict of Interest Statement

The authors declare that the research was conducted in the absence of any commercial or financial relationships that could be construed as a potential conflict of interest.

## Footnotes

1 http://www.ncbi.nlm.nih.gov

2 http://www.bioinformatics.nl/cgi-bin/primer3plus/primer3plus.cgi/

3 http://www.ebi.ac.uk/blastall/nucleotide.html
