# Supplementary material for: Plant Microbiome and Its Link to Plant Health: Host Species, Organs and Pseudomonas syringae pv. actinidiae Infection Shaping Bacterial Phyllosphere Communities of Kiwifruit Plants
Source: Front Plant Sci. 2018 Nov 7;9:1563. doi: 10.3389/fpls.2018.01563 (PMC6234494; doi:10.3389/fpls.2018.01563)

Supplementary Material

Plant Microbiome and Its Link to Plant Health: Host Species, Organs and *Pseudomonas syringae* pv. *actinidiae* Infection Shaping Bacterial Phyllosphere Communities of Kiwifruit Plants

Authors' names:

Witoon Purahong[1], Luigi Orrù[2], Irene Donati[3], Giorgia Perpetuini[3], Antonio Cellini[3], Antonella Lamontanara[2], Vania Michelotti[2], Gianni Tacconi[2] and Francesco Spinelli[3*]

Affiliations:

[1]Department of Soil Ecology, Helmholtz Center for Environmental Research - UFZ, Halle, Germany

[2]CREA Research Centre for Genomics and Bioinformatics - Fiorenzuola d'Arda, Italy

[3]Department of Agricultural and Food Sciences, Alma Mater Studiorum – Università di Bologna, Bologna, Italy

Figure S1. (A) Rarefaction curves of bacterial OTUs in leaves and flower samples. Ac: *Actinidia chinensis*, Ad: *Actinidia deliciosa*; DF: diseased flowers; DL: diseased leaves; HF: healthy flowers; DL: diseased leaves. Blue lines above and below indicate the 95% confidence. Bacterial OTUs richness values at 6,886 sequences per sample are shown above the individual rarefaction curves. (B) Melting curve relative to primer sets used for the quantification of *Pseudomonas syringae* pv. *actinidiae* and (C) *Lactobacillus plantarum*.

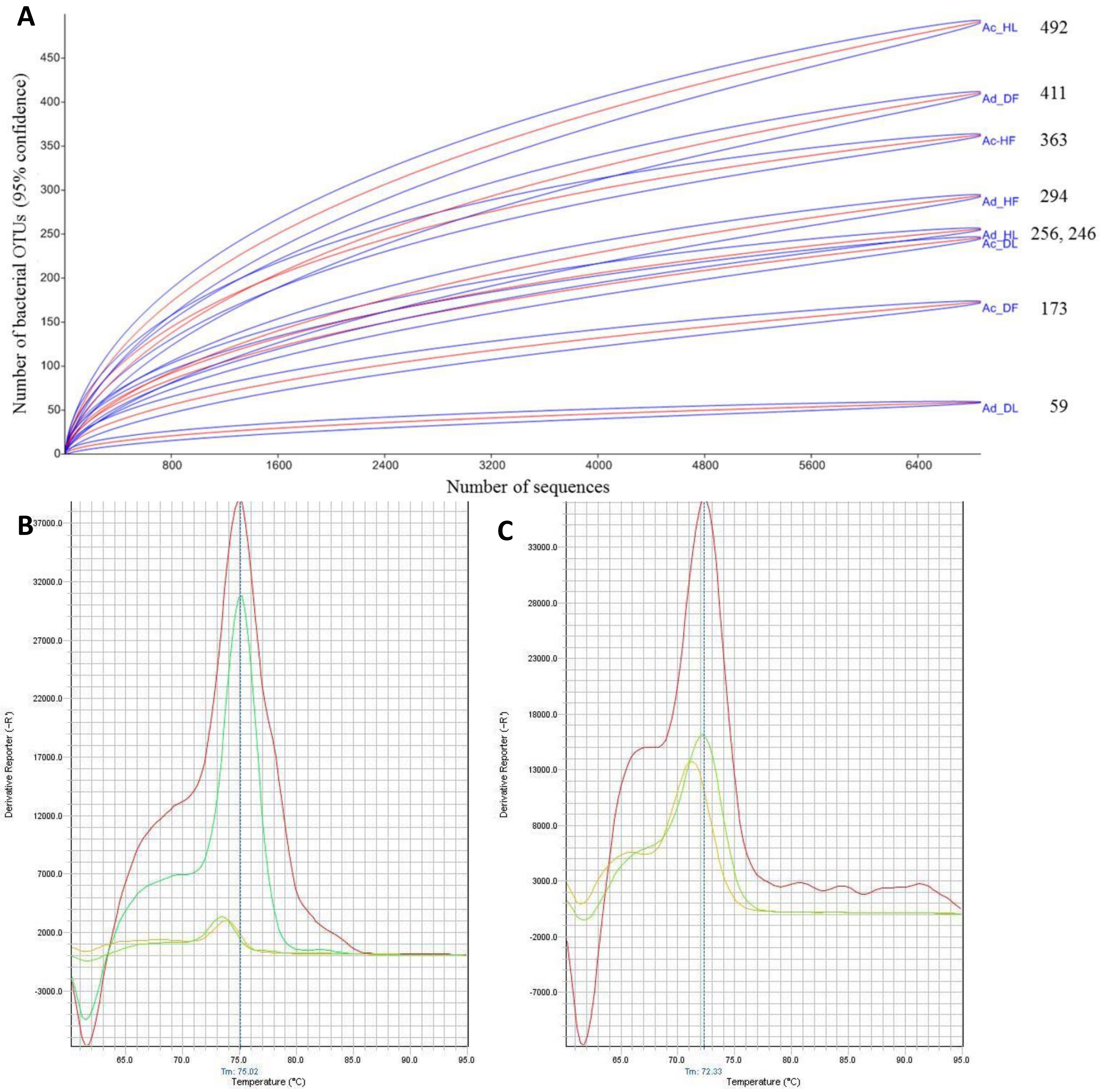

Supplement: Supplementary file 1 [file Presentation_1.pdf]
